# Ultraplex: A rapid, flexible, all-in-one fastq demultiplexer

**DOI:** 10.12688/wellcomeopenres.16791.1

**Published:** 2021-06-07

**Authors:** Oscar G Wilkins, Charlotte Capitanchik, Nicholas M. Luscombe, Jernej Ule

**Affiliations:** 1 The Francis Crick Institute, London, UK; 2 Department of Neuromuscular Diseases, UCL Queen Square Institute of Neurology, London, UK; 3 UCL Genetics Institute, Department of Genetics, Environment and Evolution, University College London, London, UK; 4 Okinawa Institute of Science & Technology Graduate University, Okinawa, Japan

**Keywords:** Demultiplexing, fastq, iCLIP, UMI, ribosome profiling

## Abstract

**Background: **The first step of virtually all next generation sequencing analysis involves the splitting of the raw sequencing data into separate files using sample-specific barcodes, a process known as “demultiplexing”. However, we found that existing software for this purpose was either too inflexible or too computationally intensive for fast, streamlined processing of raw, single end fastq files containing combinatorial barcodes.

**Results:** Here, we introduce a fast and uniquely flexible demultiplexer, named Ultraplex, which splits a raw FASTQ file containing barcodes either at a single end or at both 5’ and 3’ ends of reads, trims the sequencing adaptors and low-quality bases, and moves unique molecular identifiers (UMIs) into the read header, allowing subsequent removal of PCR duplicates. Ultraplex is able to perform such single or combinatorial demultiplexing on both single- and paired-end sequencing data, and can process an entire Illumina HiSeq lane, consisting of nearly 500 million reads, in less than 20 minutes.

**Conclusions:** Ultraplex greatly reduces computational burden and pipeline complexity for the demultiplexing of complex sequencing libraries, such as those produced by various CLIP and ribosome profiling protocols, and is also very user friendly, enabling streamlined, robust data processing. Ultraplex is available on PyPi and Conda and via Github.

## Introduction

Next generation sequencing (NGS) has greatly reduced the cost of obtaining large amounts of sequence data, as hundreds of millions, or even billions, of reads can be generated in a single sequencing run ( Goodwin *et al.*, 2016). However, despite a greatly reduced cost per read, the cost of each sequencing run is still high. To reduce the cost per sample, a single sequencing run will typically involve multiplexing of multiple samples. To enable identification of which sample a given read is derived from, sample-specific “barcodes” (short, defined DNA sequences) are introduced during library preparation. Following sequencing, software is required to detect these barcodes and split the reads into separate files. Only after demultiplexing can read alignment and other downstream analysis be performed.

**Figure 1.**
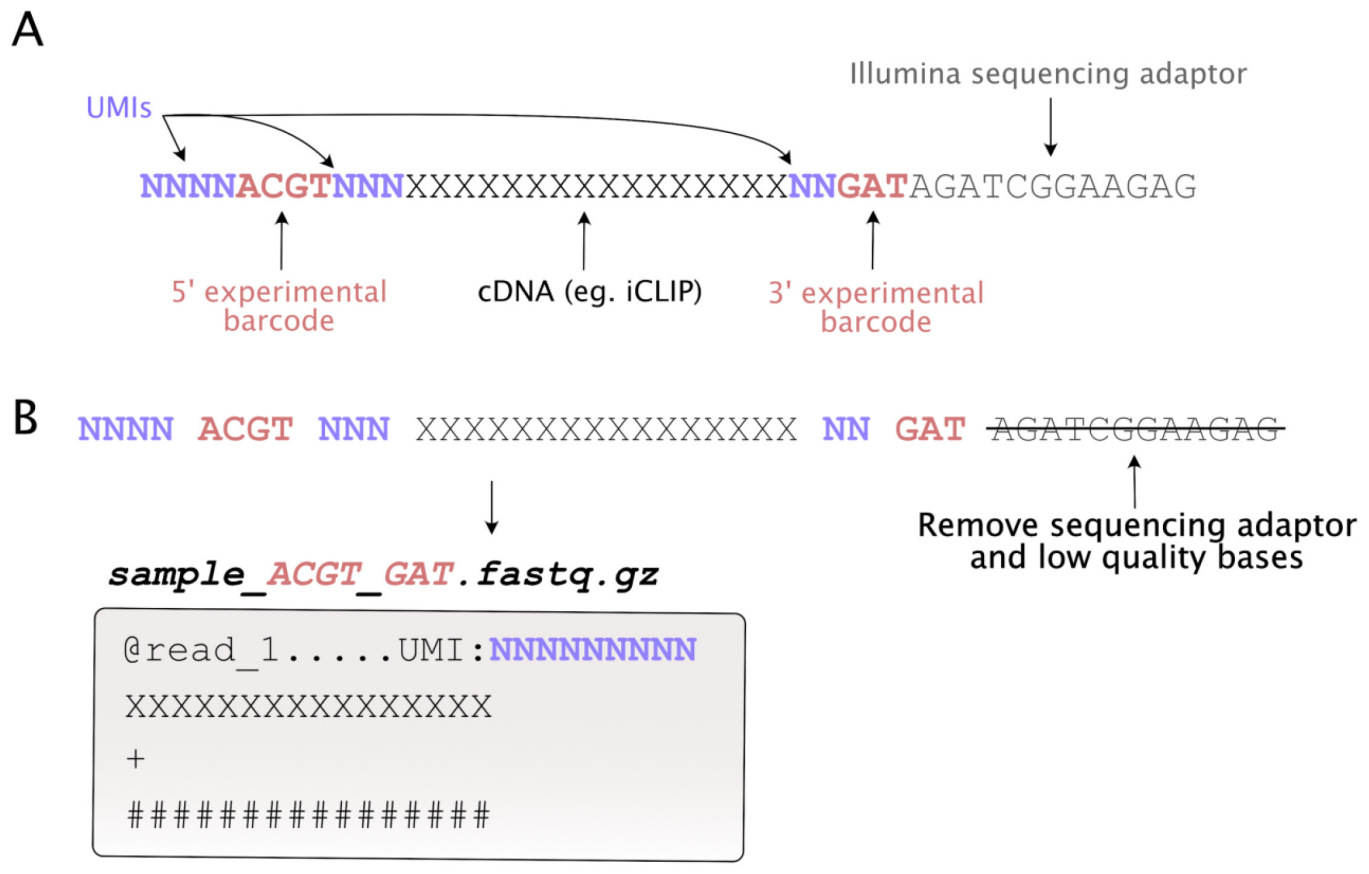
The Ultraplex workflow. **A**: An example read from a typical iCLIP/ribo-seq library, consisting of twin barcodes, UMIs present at multiple positions, a 3’ sequencing adaptor, and a read derived from a small RNA fragment. **B**: Flow diagram of the processing of an example read, using combinatorial demultiplexing with single end sequencing data.

For commercial library preparation methods (for example, Lexogen Quant-seq or Illumina Truseq), demultiplexing is typically performed during the generation of fastq files from the raw read data. For Illumina sequencing, the software used for this is Bcl2fastq. However, many in-house library preparation protocols use custom barcodes that are introduced via adaptors in such a way that they are present at 5’ and/or 3’ ends of reads, such as iCLIP (individual nucleotide crosslinking and immunoprecipitation) ( Huppertz *et al.*, 2014; König *et al.*, 2010) or related protocols to study protein-RNA interactions and RNA methylation ( Lee & Ule, 2018), as well as ribosome profiling and many others ( Sugimoto *et al.*, 2015). In such cases of ‘complex multiplexed libraries’, demultiplexing is typically performed at a later stage, using a fastq file consisting of all the raw reads as input. In addition to barcodes, iCLIP-style reads contain unique molecular identifiers (UMIs), which enable removal of PCR duplicates and may be spread across multiple positions in the read ( König *et al.*, 2010; Smith *et al.*, 2017; Figure 1A). Furthermore, combinatorial barcoding may be used, where each sample is identified by a unique combination of 5’ and 3’ barcodes. This allows more samples to be multiplexed on a single lane, can reduce technical variation by enabling earlier mixing of samples and enables incorporation of extra UMI nucleotides to increase UMI complexity, thus reducing the chance of UMI saturation at signal peaks ( Blazquez *et al.*, 2018).

Over the last decade, great effort has been made to improve the accuracy and speed of demultiplexing algorithms ( Aronesty, 2013; Kong, 2011; Lab, 2014; Liu, 2019; Martin, 2011; Murray & Borevitz, 2018; Roehr *et al.*, 2017; Schubert *et al.*, 2016). However, despite the large number of software packages being available for demultiplexing, we found that only iCount demultiplex ( König *et al.*, 2010) was capable of demultiplexing lanes featuring experimental barcodes split over the 5’ and 3’ of single end reads and additionally allowing that different 5’ barcodes may have different sets of accompanying 3’ barcodes ( Table 1). For such libraries, using any of the other available options would require the demultiplexer to be run multiple times, with different settings for each intermediate file, increasing time and pipeline complexity. While the iCount demultiplex algorithm offers the greatest flexibility, it is limited by speed (a full lane can take more than eight hours to process), which presents a significant bottleneck in the analysis pipeline. Others have used Flexbar for demultiplexing iCLIP data ( Busch *et al.*, 2020); however, Flexbar is unable to perform combinatorial 5’ and 3’ demultiplexing on single end data, and does not allow different 5’ barcodes to be associated with different sets of 3’ barcodes.

**Table 1.** A comparison of feature sets of various demultiplexers.

Software	Combinatorial demultiplexing for both single- and paired-end data	Unique 3’ barcodes for each 5’ barcode	Remove adaptors	Quality trim	Move UMIs to read header	Multi-threaded
**Cutadapt**	No	No	Yes	Yes	Yes	Yes
**Demultiplex**	No	N/A	No	No	No	No
**Flexbar 3.0**	No	No	Yes	Yes	Yes	Yes
**FASTX-Toolkit**	No	N/A	Yes	Yes	No	No
**Btrim**	No	Yes	Yes	Yes	No	No
**Axe**	No	Yes	No	No	No	No
**deML**	No	Yes	No	No	No	No
**AdaptorRemoval2**	No	Yes	Yes	Yes	No	Yes
**iCount demultiplex**	Yes	Yes	Yes	No	Yes	No
***Ultraplex***	*Yes*	*Yes*	*Yes*	*Yes*	*Yes*	*Yes*

We set out to create a demultiplexer suitable for processing the types of reads found in complex multiplexed libraries, without the caveats of existing software. Importantly, we wanted this software to run as quickly and efficiently as possible. We therefore required fully multithreaded operation, to take advantage of modern CPU architectures, and all processing to be performed in a single read-write cycle, so as to avoid read/write bottlenecks. By testing it on iCLIP libraries, we demonstrated that the resulting software, Ultraplex, meets all of these requirements. Thus, Ultraplex has broad applicability by greatly reducing the processing time for complex multiplexed libraries.

## Methods

### Implementation

**Figure 2.**
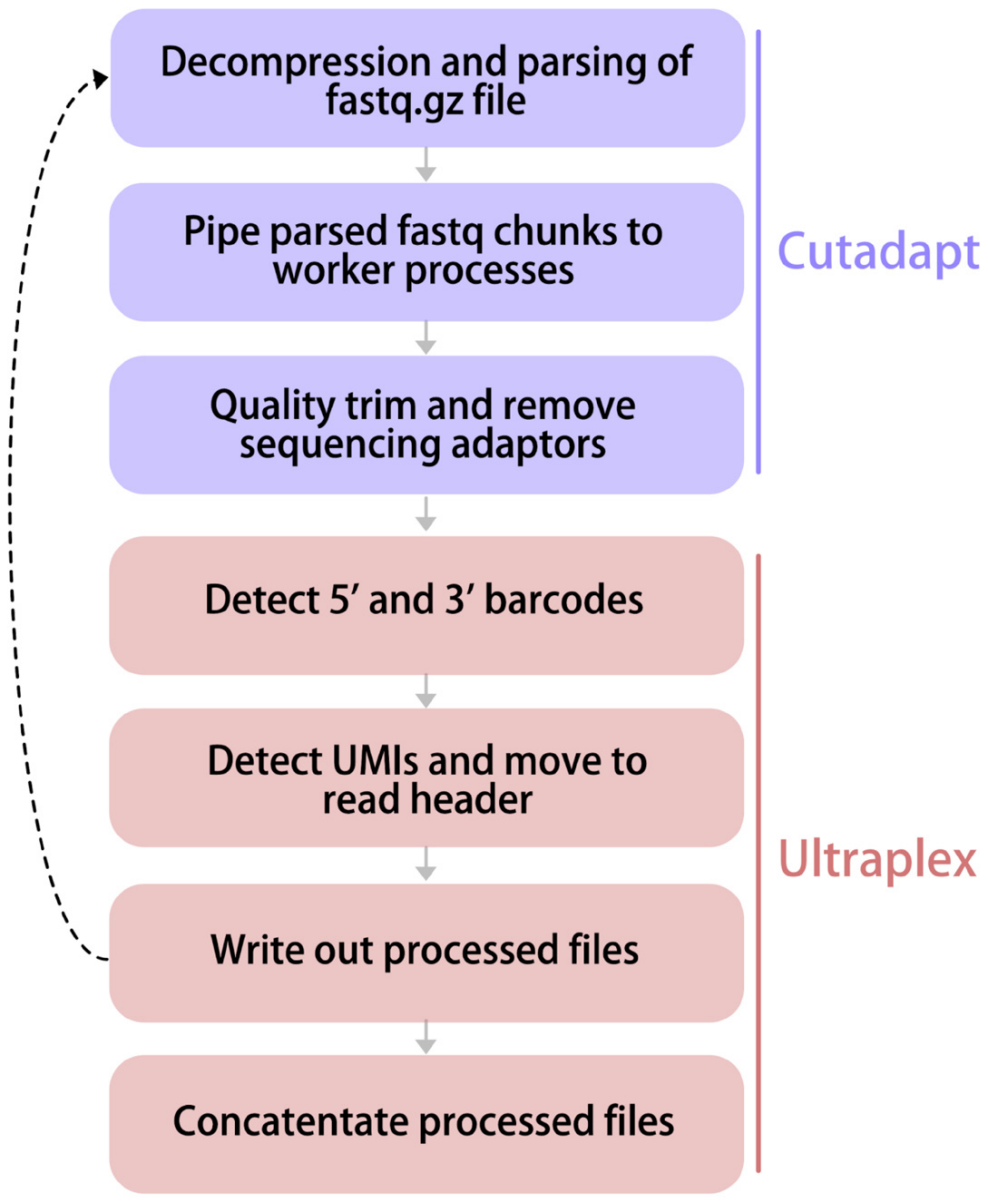
The Ultraplex pipeline. The major steps of the Ultraplex pipeline are outlined. Steps that use modified code based on Cutadapt are indicated.

Our software needed to be efficient, multithreaded and capable of performing all desired processing steps in a single read-write cycle ( Figure 1B). To this end, we utilised the high performance fastq decompression and parsing of dnaio and Cutadapt, and also used its reader/worker implementation and quality/adapter trimmer cython functions ( Martin, 2011). However, we developed bespoke solutions to demultiplexing, UMI detection and the writing of processed fastqs to enable fully multithreaded operation (at the time of writing cutadapt demultiplexing was single-threaded only), and allow more flexible demultiplexing ( Figure 2).

NGS data typically consists of hundreds of millions of reads. For efficient performance, it is therefore essential to minimise the number of function calls required for each read that is processed. For this reason, Ultraplex first generates all possible DNA sequences (including those with undefined “N” bases) of the same length as the barcodes (ignoring UMIs), then tests each sequence against each user-defined barcode to find the best matches (reads with more than one best match are discarded). By storing these precalculated best matches in a python dictionary, each read can be matched to its correct barcode or barcode pair at approximately O(1) efficiency. Typically barcodes are <= 5 bases, meaning the sequence-barcode best match function is called at most 3,125 (5 ^5^) times during dictionary generation, rather than 10 ^8^–10 ^9^ times if barcode matches were calculated for each read individually, as was the case in iCount demultiplexer.

**Figure 3.**
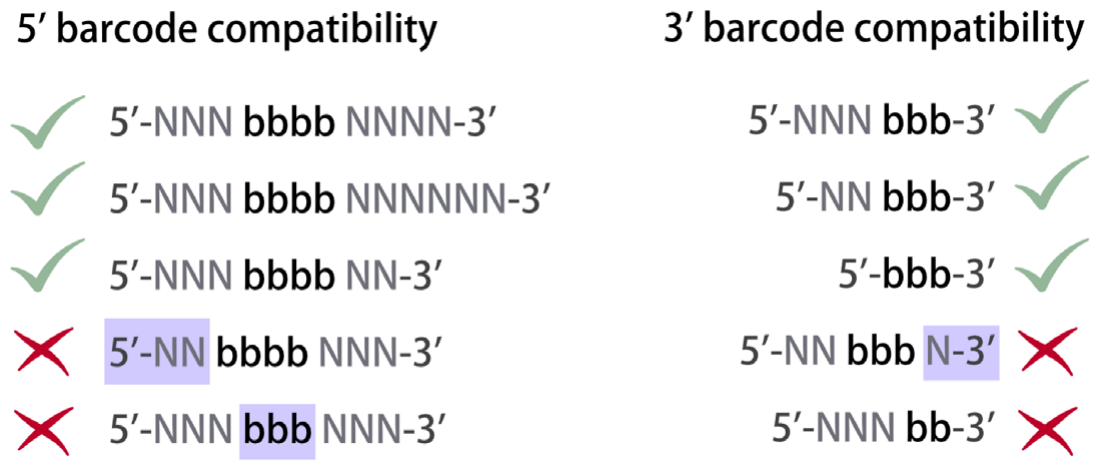
Examples of compatible and incompatible barcodes. “N” bases are randomers which would typically be used as UMIs. “b” bases are barcode bases. Compatible barcodes must have the same number of “b” bases, which must be at the same position relative to the 5’ or 3’ end of the read for 5’ and 3’ barcodes, respectively. Blue boxes indicate problematic regions of barcode.

Current multiplexing approaches use barcodes of the same type (i.e. 5’ or 3’), of consistent length, with 5’ or 3’ barcodes present at the same position within the read relative to the 5' or 3' end, respectively ( Figure 3). Such consistent design of multiplexing is important to ensure that all reads have mutually exclusive barcodes. We designed Ultraplex to enable flexible demultiplexing of any complex libraries that follow these described prerequisites. For data in which barcodes may be at unknown positions, however, alternative algorithms are required.

Ultraplex allows UMIs of different barcodes to vary in length ( Figure 3). It optionally allows each 5’ barcode to be paired with an array of 3’ barcodes, provided these 3’ barcodes are consistent, but 3’ barcodes linked to different 5’ barcodes *do not* need to be consistent with each other. All other existing demultiplexers would require multiple runs for such complex demultiplexing, increasing pipeline complexity and run-time, and therefore Ultraplex increases the flexibility, convenience and speed of demultiplexing.

When single end sequencing is used with samples containing 3’ barcodes, only cDNAs which are short enough will contain the 3’ barcode in the sequencing read (e.g. inserts of maximal length of ~90 nt will be possible for SR100, depending on barcode length). Ultraplex allows for combinatorial demultiplexing with single end data (with the above caveats), which is not possible in most other demultiplexers ( Table 1). To reduce the likelihood of erroneous detection of 3’ barcodes during single end operation, Ultraplex requires by default that at least three nucleotides of the 3’ sequencing adaptor are detected and trimmed for a 3’ barcode to be assigned; this ensures that the end of the forward read genuinely corresponds to the end of the insert, and should thus contain a 3’ barcode. Moreover, it can also demultiplex paired end sequences in which a 3’ barcode is present at the 5’ end of the reverse read, with the forward and reverse reads stored in separate FASTQ files. Ultraplex uses the forward read to detect the 5’ barcode, and the reverse read to detect the 3' barcode.

We envisage that most users of Ultraplex will run the software on a high-performance computing cluster (HPCC). HPCCs typically have large amounts of free storage space and have many separate computational nodes, on which multiple jobs can be run in parallel. To take advantage of this, we added two additional running modes, "ultra", which writes uncompressed temporary files and then compresses after concatenation, and "sbatch compression", which uses SLURM to send each compression job to a separate HPCC node. As such, the sbatch compression mode can only be used in conjunction with ultra mode, and can only be run on HPCCs with SLURM job management. The use of these two modes reduces run time by a further ~30%. These combined improvements bring an >40x increase in speed as compared to iCount, currently the only alternative tool for single-step demultiplexing of single end sequencing libraries featuring combinatorial barcodes.

### Operation

*Ultraplex* is a command-line tool which can be installed via pip or conda. It requires at least two input arguments: a comma-separated values (.csv) file of barcodes, and a compressed fastq file. A simple command would be:


ultraplex -b my_barcodes.csv -i my_fastq.fastq.gz


The first column of the barcode csv file should correspond to the 5’ barcodes; additional columns (separated by commas) correspond to any 3’ barcodes which are linked to the 5’ barcode. Optionally, each barcode can be assigned a sample name using a colon spacer (5’ barcodes cannot be assigned a sample name if linked to 3’ barcodes, as this would be ambiguous). N characters denote positions that correspond to UMIs. An example barcode csv could be as follows:


NNNATGCNN
NNNATTANNN:sample_2
NNNGCGGN,NNAA:sample_3,NNNTT


This barcode csv corresponds to four samples: two with only a 5’ barcode, and two with a shared 5’ but unique 3’ barcode. Only samples 2 and 3 are explicitly named. Note the consistency of the positioning of barcodes relative to the 5’ or 3’ ends ( Figure 3).

There are many optional arguments: -d (output directory for files), -m5 and -m3 (the number of mismatches allowed during 5’ and 3’ barcode detection), -q (the minimum phred quality during 3’ quality trimming), -t (number of threads used during operation), -a (the 3’ adaptor for the forward read), -o (an output filename prefix), -sb (sbatch compression for slurm clusters), -u (ultra mode, described above), -l (the minimum length of the read to be written out), -i2 (a second fastq for paired-end demultiplexing), -a2 (the sequencing adaptor to be trimmed for the reverse read), -inm/--ignore_no_match (does not write out reads which are not matched to sample).

## Results

**Figure 4.**
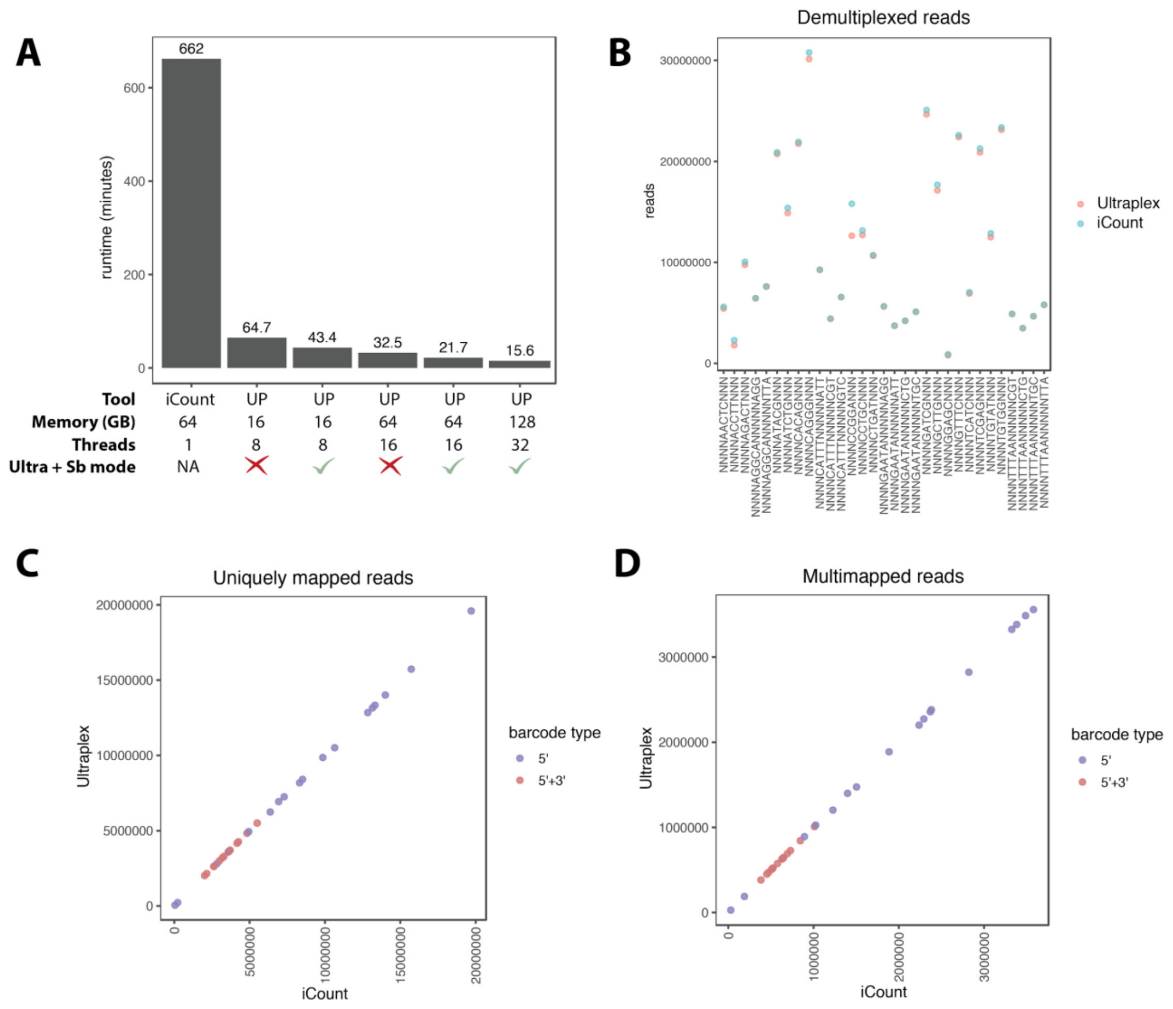
Performance of Ultraplex vs. iCount demultiplex. **A**: Runtime in minutes of iCount vs. Ultraplex (UP) with various parameters. **B**: Number of demultiplexed reads per barcode assigned by Ultraplex and iCount. **C**, **D**: Number of uniquely mapped and multimapped reads per sample after STAR mapping of the 30 iCLIP samples demultiplexed using Ultraplex or iCount.

We benchmarked Ultraplex against iCount for a sequencing lane of 482,988,240 single end reads, consisting of 30 multiplexed iCLIP samples, where 17 have only a 5’ barcode, and the remainder have both 5’ and 3’ barcodes. Our testing was run on a high-performance computing cluster where each CPU node is an 8-core Intel E5-2640 Haswell CPU running at 2.6GHz, with hyperthreading enabled (two threads per core), running Linux 3.10.0–957.1.3.el7.x86_64. iCount was run with additional flags -- min_adapter_overlap 3 -mis 1 -ml 0 and Ultraplex with -mt 3 -m5 1 -q 0 -l 17. Using Ultraplex with both ultra and sb modes, 16 threads and 64GB memory, the lane was demultiplexed in 21.7 minutes, but we could push this as low as 15.6 minutes by allowing 32 threads and 128GB memory ( Figure 4A). Given 64GB memory and matched settings, iCount took 662 minutes (~ 11 hours). Even without ultra and sb modes enabled, Ultraplex only took 32.5 minutes. We also tested Ultraplex with lower resources; given eight threads and 16GB memory, the lane was demultiplexed in 43.4 minutes with ultra and sb modes enabled, and 64.7 minutes without. This demonstrates that even with modest resource allocation, Ultraplex is a very fast demultiplexer.

Next we compared the output of iCount and Ultraplex to check consistency. Reassuringly, Ultraplex gave exactly the same results over the four different test runs. Comparing iCount to Ultraplex, the number of reads assigned per barcode were mostly the same, bar a few samples where iCount assigned slightly more reads ( Figure 4B). To explore this, we further filtered reads with cutadapt quality trimming removing 3’ nucleotides with PHRED score of less than 30, kept reads with a minimum length of 20 nucleotides, and mapped them to the human genome using STAR. The biggest deviance between iCount and Ultraplex assigned reads was found for the sample NNNNCCGGANNN. For this sample, only 0.99% (2090/211347) of the iCount-specific reads mapped to the genome, and 90% (1898/2090) of these were assigned as “spliced”, meaning the read had to be split to be mapped, indicative of low-quality mapping. The remaining 99.01% of the iCount-specific reads were determined by STAR to be too short to map. When comparing the total number of mapped reads for all samples (both unique and multi-mapped), we found the final results of Ultraplex and iCount were near-identical ( Figure 4C,D). Thus, Ultraplex produces essentially identical results to iCount, but is >40x faster.

## Conclusions

The processing of iCLIP-style sequencing libraries consists of many sequential steps, which requires complex pipelines ( Busch *et al.*, 2020; Chakrabarti *et al.*, 2018). By performing multiple processing steps in one read/write cycle, and by using a multi-threaded and computationally efficient method, Ultraplex greatly improves the speed and ease of the initial steps of demultiplexing the fastq file. In our testing we find Ultraplex to be up to 40 times faster than the currently used iCount software. Furthermore, Ultraplex allows for extremely flexible demultiplexing, simplifying the analysis when multiple samples with varying barcode arrangements are sequenced together. By removing the largest time bottleneck in the CLIP analysis workflow, we now make it possible to go from multiplexed fastq to sample crosslinks in a matter of hours using a pipeline such as nf-core/clipseq ( Ewels *et al.*, 2020).

## Data availability

### Underlying data

ArrayExpress: Ultraplex: An ultra-fast, flexible, all-in-one fastq demultiplexer. Accession number E-MTAB-10349; https://identifiers.org/arrayexpress:E-MTAB-10349.

## Software availability

Source code available from: https://github.com/ulelab/ultraplex

Archived source code at time of publication: https://doi.org/10.5281/zenodo.4651285 ( Wilkins *et al.*, 2021)

License: MIT

Software is also on PyPi and Bioconda.
