# Supplementary material for: Ultraplex: A rapid, flexible, all-in-one fastq demultiplexer
Source: Wellcome Open Res. 2021 Jun 7;6:141. [Version 1] doi: 10.12688/wellcomeopenres.16791.1 (PMC8287537; doi:10.12688/wellcomeopenres.16791.1)
Supplement: Peer review history

# Peer review: Reviewer response for version 1

**DOI:** 10.21956/wellcomeopenres.18522.r44297

**Published:** 2021-07-16

**Authors:** Grzegorz Kudla

**Affiliations:** 1 MRC Human Genetics Unit, Institute for Genetics and Molecular Medicine, The University of Edinburgh, Edinburgh, UK

Wilkins *et al. *describe a new software tool, called Ultraplex, for demultiplexing of fastq files. Demultiplexing is an essential data preprocessing step in the analysis of various functional genomic experiments, including CLIP, iCLIP, eCLIP, PAR-CLIP, CRAC, Ribo-Seq, CLASH, and others.

 Ultraplex inputs sequencing data in fastq format, and it outputs fastq files that have been separated using the barcode (index) information included in reads. The tool is flexible: it can process split barcodes distributed between 5' and 3' ends of reads; it processes UMI information to facilitate the removal of PCR duplicates; it allows processing of single- and paired-end data; and, thanks to the integration of the cutadapt tool, it allows trimming of adapter sequences and low quality nucleotides. As such, ultraplex can facilitate data analysis by running the entire data preprocessing as a single command.

 I found the tool easy to install using pip, and intuitive to run. There is extensive documentation on github. The performance and outputs are as described in the manuscript. In future updates, it would be useful to add a functionality to allow processing of barcodes of different lengths, since those are sometimes used in the same run to increase nucleotide diversity in each cycle of sequencing.

Are the conclusions about the tool and its performance adequately supported by the findings presented in the article?

Yes

Is the rationale for developing the new software tool clearly explained?

Yes

Is the description of the software tool technically sound?

Yes

Are sufficient details of the code, methods and analysis (if applicable) provided to allow replication of the software development and its use by others?

Yes

Is sufficient information provided to allow interpretation of the expected output datasets and any results generated using the tool?

Yes

Reviewer Expertise:

RNA biology, RNA-protein interactions, RNA-RNA interactions

I confirm that I have read this submission and believe that I have an appropriate level of expertise to confirm that it is of an acceptable scientific standard.

# Peer review: Reviewer response for version 1

**DOI:** 10.21956/wellcomeopenres.18522.r44299

**Published:** 2021-06-18

**Authors:** Matteo Cereda

**Affiliations:** 1 Cancer Genomics and Bioinformatics Unit, IIGM-Italian Institute for Genomic Medicine, Turin, Italy

In this work, Wilkins *et al.* provide a fast and flexible multithreaded demultiplexer that can be applied to many NGS approaches solving the complex issue of combinatorial demultiplexing. The algorithm can deal with different experimental designs optimizing the computational cost. The software comes with a well-structured repository and all functions are clearly explained.

 This Reviewer would suggest mentioning in the Introduction other examples of sequencing technologies (i.e. single cells, clonal tracking) that can take advantage of Ultraplex. 

**Minor:**

- How would the computational cost increase with barcodes longer than 5nt (up to 10 max)?

Are the conclusions about the tool and its performance adequately supported by the findings presented in the article?

Yes

Is the rationale for developing the new software tool clearly explained?

Yes

Is the description of the software tool technically sound?

Yes

Are sufficient details of the code, methods and analysis (if applicable) provided to allow replication of the software development and its use by others?

Yes

Is sufficient information provided to allow interpretation of the expected output datasets and any results generated using the tool?

Yes

Reviewer Expertise:

bioinformatics; alternative splicing; cancer genomics

I confirm that I have read this submission and believe that I have an appropriate level of expertise to confirm that it is of an acceptable scientific standard.
